# Purkinje Cell Maturation Participates in the Control of Oligodendrocyte Differentiation: Role of Sonic Hedgehog and Vitronectin

**DOI:** 10.1371/journal.pone.0049015

**Published:** 2012-11-14

**Authors:** Lamia Bouslama-Oueghlani, Rosine Wehrlé, Mohamed Doulazmi, Xiao Ru Chen, Fanny Jaudon, Yolande Lemaigre-Dubreuil, Isabelle Rivals, Constantino Sotelo, Isabelle Dusart

**Affiliations:** 1 Neurobiologie des Processus Adaptatif, Université Pierre et Marie Curie (UPMC Univ Paris 06), Paris, France; 2 Neurobiologie des Processus Adaptatif, CNRS (Centre National de Recherche Scientifique), Paris, France; 3 Centre de Recherche de Biochimie Macromoléculaire, Université Montpellier 1 et 2, CNRS UMR 5237, Montpellier, France; 4 Équipe de statistique Appliquée, ESPCI ParisTech (Ecole Supérieure de Physique et Chimie Industrielles de la Ville de Paris), Paris, France; 5 Instituto de Neurociencias, Universidad Miguel Hernández–CSIC, San Juan de Alicante, Spain; Louisiana State University Health Sciences Center, United States of America

## Abstract

Oligodendrocyte differentiation is temporally regulated during development by multiple factors. Here, we investigated whether the timing of oligodendrocyte differentiation might be controlled by neuronal differentiation in cerebellar organotypic cultures. In these cultures, the slices taken from newborn mice show very few oligodendrocytes during the first week of culture (immature slices) whereas their number increases importantly during the second week (mature slices). First, we showed that mature cerebellar slices or their conditioned media stimulated oligodendrocyte differentiation in immature slices thus demonstrating the existence of diffusible factors controlling oligodendrocyte differentiation. Using conditioned media from different models of slice culture in which the number of Purkinje cells varies drastically, we showed that the effects of these differentiating factors were proportional to the number of Purkinje cells. To identify these diffusible factors, we first performed a transcriptome analysis with an Affymetrix array for cerebellar cortex and then real-time quantitative PCR on mRNAs extracted from fluorescent flow cytometry sorted (FACS) Purkinje cells of L7-GFP transgenic mice at different ages. These analyses revealed that during postnatal maturation, Purkinje cells down-regulate Sonic Hedgehog and up-regulate vitronectin. Then, we showed that Sonic Hedgehog stimulates the proliferation of oligodendrocyte precursor cells and inhibits their differentiation. In contrast, vitronectin stimulates oligodendrocyte differentiation, whereas its inhibition with blocking antibodies abolishes the conditioned media effects. Altogether, these results suggest that Purkinje cells participate in controlling the timing of oligodendrocyte differentiation in the cerebellum through the developmentally regulated expression of diffusible molecules such as Sonic Hedgehog and vitronectin.

## Introduction

Oligodendrocytes are central nervous system macroglial cells that synthesize myelin, a multilayered membrane ensheathing axons which facilitates rapid nerve conduction [1]. During development, oligodendrocyte precursor cells (OPCs) divide and migrate over long distances to reach their final destination where they differentiate into mature oligodendrocytes and produce myelin.

Neuron maturation affects oligodendrocyte survival and the timing of myelin formation, OPCs nonetheless differentiate into mature oligodendrocytes and generate a myelin sheath in the absence of axons in vitro [2], [3]. In the optic nerve, only the oligodendrocytes ensheathing axons survive [4], [5]. Oligodendrocytes are more abundant in transgenic mice with larger numbers of axons [6]. Myelin formation is correlated with certain parameters of axonal maturation, such as axon caliber and neurofilament content [7]–[9]. Axonal factors which are directly involved in controlling myelin formation include neuronal electrical activity [10], [11] and the downregulation of various molecules in axonal membranes, including Jagged1, PSA-NCAM (polysialic acid-neural cell adhesion molecule) and N-cadherin [12]–[14]. Myelin membrane formation is coordinated by a large number of proteins, through contact mechanisms and integrin receptors [15]. Furthermore, Rosenberg and colleagues demonstrated that myelin formation required an axonal microenvironment and a critical density of OPCs [16].

The role of neurons in the switch between OPC proliferation and differentiation into oligodendrocytes remains unclear. The timing of this switch depends on both the intracellular timer and extrinsic factors [17]. For several years, thyroid hormone (T3), retinoic acid (RA), glucocorticoids and transforming growth factor (TGFß) were the only molecules known to trigger the initial stages of OPC differentiation [18], [19]. More recently, neuronal activity has also been shown to participate in OPC differentiation. Purinergic receptor activation by non-synaptically released adenosine [20] stimulates the differentiation of OPCs into oligodendrocytes. Thus, reciprocal neuron-glial interactions are also required for the complete conversion of OPCs into differentiated oligodendrocytes. These neuron-glial interactions do not always have positive effects; connective tissue growth factor (CFTG) has been reported to inhibit the differentiation of OPCs into oligodendrocytes through interactions with serum response factor (SRF), a neuronal transcription factor [21].

In this study, we investigated the existence of neuronal soluble factors controlling oligodendrocyte differentiation in an integrated system. For that purpose, we used cerebellar organotypic cultures, in which neuron-glial interactions mimic those occurring in vivo and in which only one type of neuron, the Purkinje cell, is myelinated [22]. We demonstrated that the maturation of Purkinje cells is one of the key factors controlling the timing of oligodendrocyte differentiation. Indeed, Purkinje cells timely release two factors, Sonic Hedgehog (Shh) and vitronectin (VN), which respectively stimulate OPC proliferation and oligodendrocyte differentiation.

## Results

### Newborn cerebellar slice cultures are immature for oligodendrocyte differentiation during the first week in vitro and become mature during the second week

**Figure 1:**
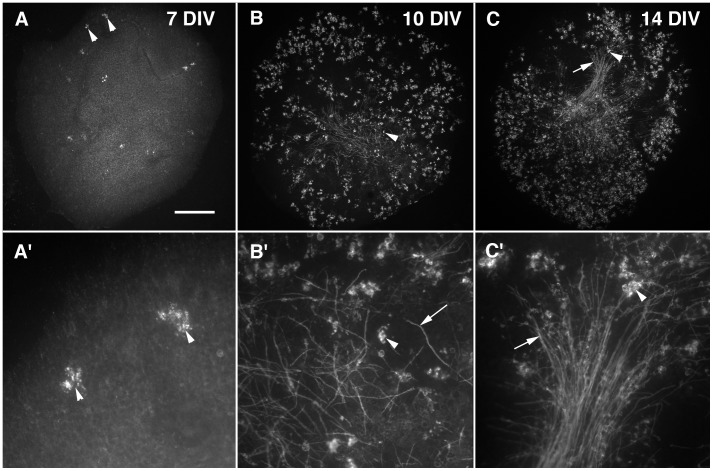
Oligodendrocyte differentiation and myelination on mouse newborn slices occurred during the second week of organotypic culture. **A-A′.** Photomicrographs of a newborn cerebellar slice maintained for 7 days in vitro (DIV) and labeled with antibodies against MBP. A′ is an enlargement of A. Note that the number of MBP immunoreactive processes of the oligodendrocytes (arrowheads) is low, indicating the immature stage of 7 DIV slices in terms of oligodendrocyte differentiation. **B-B′.** Photomicrographs of a P0 cerebellar slice maintained for 10 DIV and labeled with antibodies against MBP. B′ is an enlargement of B. The MBP immunoreative elements are composed of oligodendrocyte processes (arrowhead) and of internodes (arrow). Note that the number of MBP immunoreative oligodendrocytes is very high and that of MBP immunoreactive internodes is consequent, indicating that 10 DIV slices have reached a mature stage of 10 DIV oligodendrocyte differentiation, although myelin formation has just started. **C-C′.** Photomicrographs of P0 cerebellar slice maintained for 14 DIV and labeled with antibodies against MBP. C′ is an enlargement of C. The MBP immunostaining reveals both oligodendrocytes (arrowhead) and internodes (arrows). The numbers of MBP immunoreactive oligodendrocytes and internodes are high, indicating that both oligodendrocte differentiation and myelin formation are well advanced. Bar is 350 µm in A, B and C and 60 µm in A′, B′ and C′.

To analyze the timing of the oligodendrocyte differentiation process in cerebellar slice cultures, we focused on the expression of MBP because this protein is expressed in mature oligodendrocytes (both pre- and myelinating oligodendrocytes [1], [23]). MBP immunostaining was detected both on the processes of mature oligodendrocytes (Fig. 1A′, B′, C′) and the myelin segments (internodes, Fig. 1B′, C′). The appearance of this protein therefore reflects the last stages of oligodendrocyte differentiation.

P0 cerebellar slices grown for 7 DIV had very few MBP+ oligodendrocytes (Fig. 1A, 1A′), and very few if any MBP+ internodes were observed at this stage. Indeed, most of the slices (over 75%) did not present any internodes (Fig. 1A), and we did not detect more than 25 MBP+ internodes on the other slices. We defined Group I as the slices containing less than 25 internodes and Group II as the slices containing more than 26 internodes. Thus, on P0–7 DIV slices, the density of MBP staining can be used as an index of oligodendrocyte differentiation, since very few internodes were present.

At 10 DIV, MBP+ oligodendrocytes were present throughout the slices (Fig. 1B) and MBP+ internodes can be detected (Fig. 1B′). At 10 DIV, over 50% of the slices exhibited more than 26 internodes per slice and were therefore in Group II. The process of oligodendrocyte differentiation is well engaged, whereas myelination had only just started.

After 14 DIV, the number of MBP+ internodes increased (Fig. 1C). Almost all of the slices contained more than 26 internodes and were therefore in Group II. Furthermore, myelin tracts can often be observed (Fig. 1C′). At 14 DIV, the process of myelination is already well engaged.

Our results therefore showed that most OPCs differentiated into MBP+ oligodendrocytes between 7 and 10 DIV and most of the myelination process occurred between 10 and 14 DIV. P0 slices were thus considered to be immature for oligodendrocyte differentiation during the first 7 DIV and to be mature during the following week, in which rapid oligodendrocyte differentiation occurred.

### Mature cerebellar slices release factors that increase oligodendrocyte differentiation in immature slices

**Figure 2:**
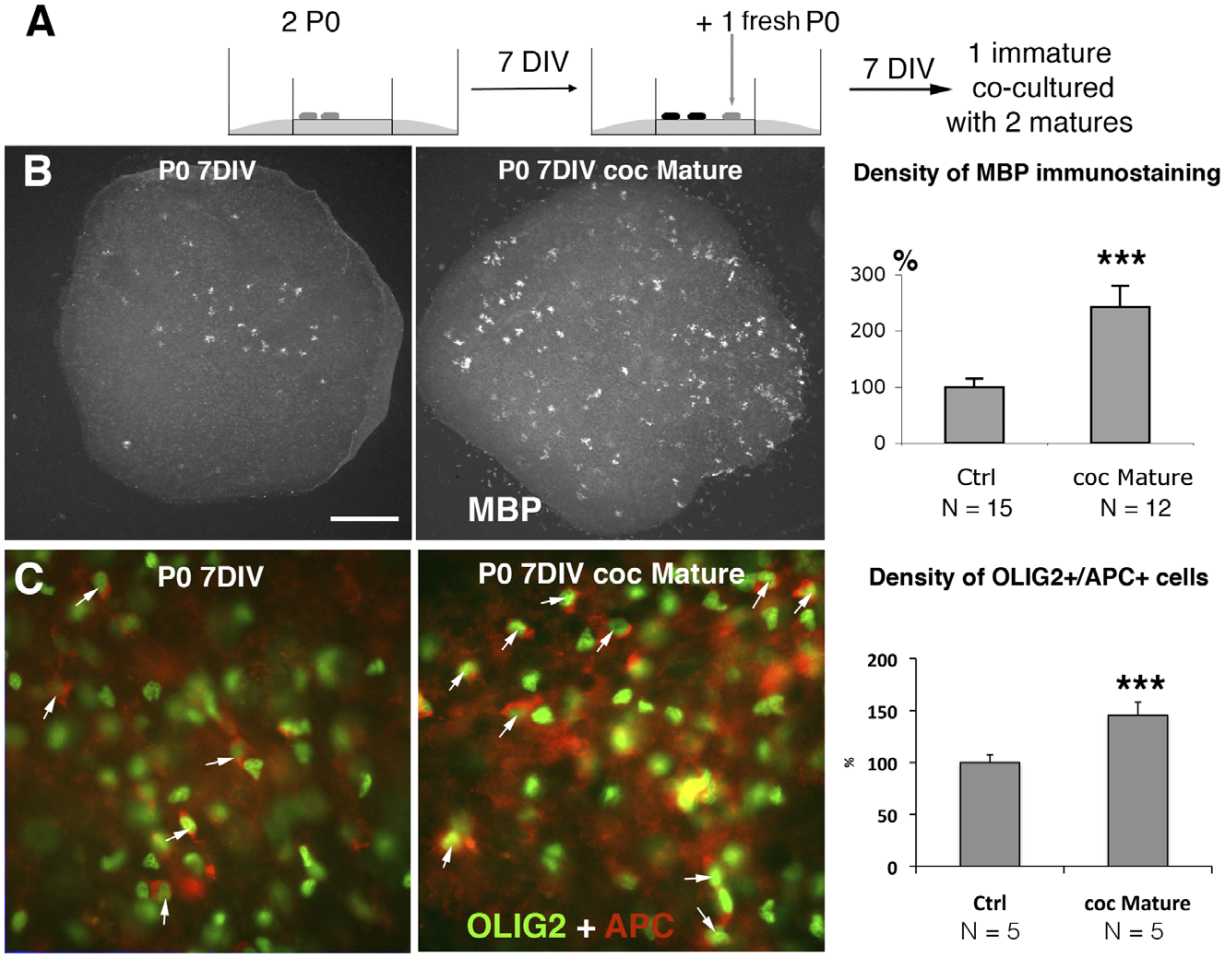
Mature slices increase the density of MBP+ oligodendrocytes in immature slices, but not of OLIG2+ cells. **A.** Schematic diagram of the coculture experiment: 2 P0 cerebellar slices (gray) were plated. After 7 DIV, they became mature (black) and one fresh P0 cerebellar slice (gray) was added to the same Millicell, which was incubated for a further 7 days. **B.** Photomicrographs of P0 cerebellar slices maintained until 7 DIV and labeled with antibodies against MBP. There are fewer MBP+ elements in the control slice (B, P0–7 DIV) than in the P0–7 DIV immature slice co-cultured in the presence of 2 P0 7–14 DIV mature slices (P0–7 DIV coc Mature). Quantitative evaluation of the density of MBP immunostaining in P0–7 DIV immature slices (Ctrl) and P0–7 DIV immature slices co-cultured in the presence of P0 7–14 DIV mature slices (coc Mature). *** P<0.001 (Mann-Whitney test). **C.** Mature slices increase the density of OLIG2+/APC+ oligodendrocytes in immature slices: Photomicrographs of P0 cerebellar slices maintained until 7 DIV and labeled with antibodies against OLIG2 (green) and APC (red). There are fewer OLIG2+/APC+ (stars) cells in the control slice (P0–7 DIV) than in the P0–7 DIV immature slice co-cultured in the presence of 2 P0 7–14 DIV mature slices (P0–7 DIV coc Mature). Quantitative evaluation of the number of OLIG2+/APC+ cells in P0–7 DIV immature slices (Ctrl) and P0–7 DIV immature slices co-cultured in the presence of P0 7–14 DIV mature slices (coc Mature). *** P<0.001 (Mann-Whitney test). Scale bar represents 320 µm in B and 35 µm in C.

We investigated whether, during the phase of oligodendrocyte differentiation, cells in the slice culture (between 7 and 14 DIV) were able to increase oligodendrocyte differentiation on immature slices (7 DIV). To this aim, P0 cerebellar slices were grown in culture until 7 DIV, when the differentiation of oligodendrocytes was beginning and could be called “mature”. At this time point, fresh P0 slices (immature) were added to the Millicell (Fig. 2A) such that the ratio of immature/mature slices was 1∶2. After 7 more DIV, we analyzed the MBP immunostaining on the immature slices (P0–7DIV control (ctrl) or grown in presence of mature slices (coc Mature)). We did not detect any increase in the presence of internodes; all of the P0–7 DIV slices (control slices or slices grown in the presence of mature slices) were in Group I (i.e. presenting less than 25 internodes). The MBP+ oligodendrocytes were evenly distributed in the slices (Fig. 2B). The density of MBP immunostaining was 2.44 times higher in immature slices grown in the presence of mature slices (P0–7 DIV Coc-Mature) compared with control immature slices (P<0.001, Fig. 2B).

Then to confirm that this increase of MBP immunostaining was indeed due to an increase of the number of oligodendrocytes, we evaluated the density of APC+/OLIG2+ cells on the slice cultures. APC is expressed by oligodendrocytes and some astrocytes [24], [25], whereas OLIG2+ is expressed by both OPCs and oligodendrocytes [26]. Thus, APC+/OLIG2+ cells are oligodendrocytes. We found a significant increase in the number of OLIG2+/APC+ cells in 7 DIV slices grown in presence of mature slices compared to 7 DIV control slices (Fig. 2C).

These results showed that mature slices release factors that increase oligodendrocyte differentiation in immature ones. They also suggest that the switch from OPC proliferation to oligodendrocyte differentiation in cerebellar organotypic cultures is controlled by factors released from cerebellar cells at critical stages of maturity. Then we investigated whether Purkinje cells might influence the timing of oligodendrocyte differentiation in cerebellar slices. Indeed the number of Purkinje cells can be manipulated during the first week in vitro, i.e. before the addition of immature slices.

### The oligodendrocyte differentiation effect of mature slices is proportional to the number of Purkinje cells

**Figure 3:**
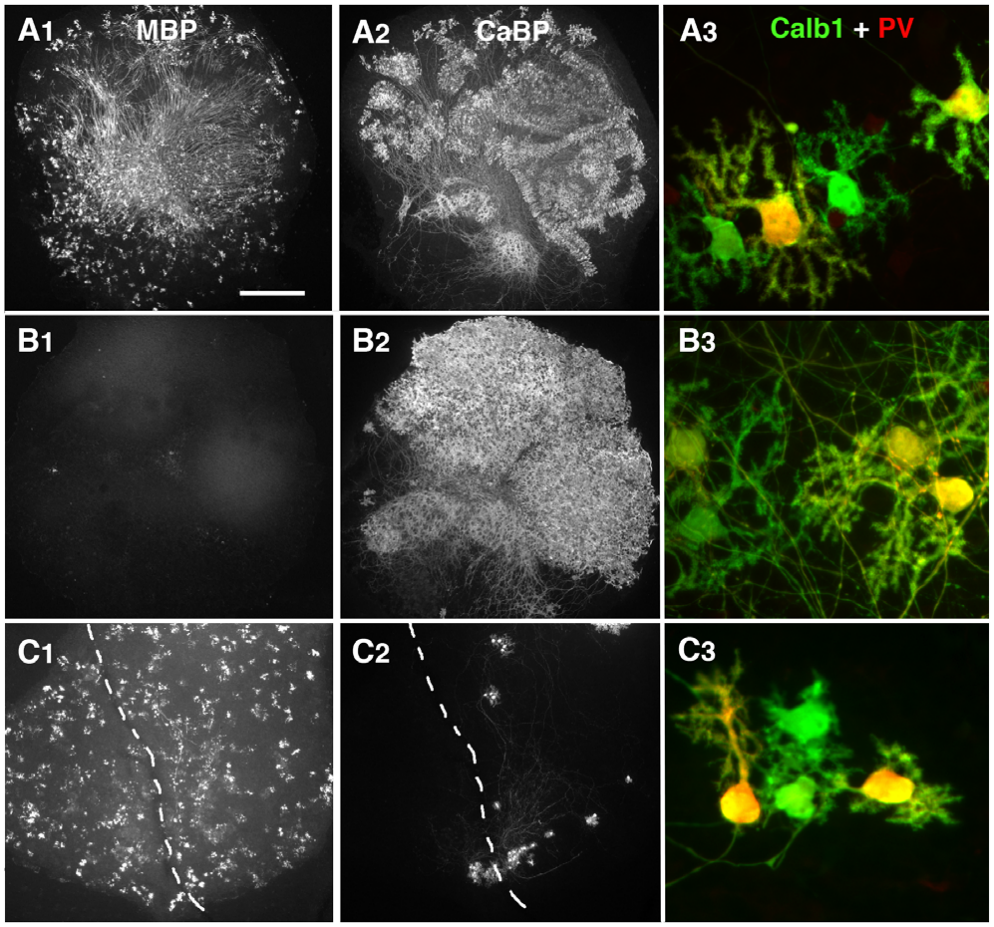
Slice culture treatments allowing drastic modifications of Purkinje cell and oligodendrocyte numbers. Photomicrographs of P0–14 DIV cerebellar slices double-labeled with antibodies against MBP (A1, B1, and C1) and Calbindin (Calb1 = CaBP; A2, B2, and C2) and double-labeled with antibodies against Calb1 (green) and Parvalbumin (PV, red) for A3, B3, and C3. A. P0–14 DIV control slice: note the presence of numerous MBP+ internodes (A1) and Calb1/CaBP positive Purkinje cells (A2). B. P0 cerebellar slice treated during the first 3 days with a high dose of BrdU. Note the absence of MBP staining (B1) and the high density of Calb1+ Purkinje cells (B2). C. The P0 slices were axotomized after 2 DIV (dotted line represent the lesion). The resulting density of Purkinje cells (C2) is much lower than that under the conditions for A and B. In all 3 conditions (A3, B3, and C3), Purkinje cell were differentiated since some of them presented elaborated dendritic tree with spines and expressed parvalbumin (yellow Purkinje cells). Scale bar is 390 µm for A1, B1, C1, A2, B2, and C2 and 30 µm in A3, B3, and C3.

To investigate whether Purkinje cells might control the secretion of these differentiation factors, we used two different slice-culture models in which the numbers of Purkinje cells were markedly different. To increase the number of Purkinje cells, we applied a BrdU treatment that kills dividing cells during the first 3 DIV of the culture [27]. In this model, after 14 DIV, the BrdU-treated slices contained no MBP+ elements (Fig. 3B1) and much more Purkinje cells than the P0–14DIV control slices (compare Figs. 3B2 with 3A2).

To decrease the number of Purkinje cells, we took advantage of the fact that newborn Purkinje cells axotomized after 2 DIV, die in large numbers in organotypic culture. Indeed, axotomized slices contained small numbers of surviving Purkinje cells in the second week of culture (Fig. 3C2). These axotomized slices contained some MBP+ oligodendrocytes (Fig. 3C1).

We then evaluated whether the Purkinje cells have reached a differentiated stage at 14 DIV in all 3 different conditions (Ctrl, BrdU-treated slices or axotomy-treated slices).

We previously showed that at the time of the culture, newborn Purkinje cells were bipolar, whereas after 7 DIV, most Purkinje cells have retracted their primitive processes and developed numerous perisomatic protrusions [28], [29]. After 14 DIV, Purkinje cells presenting two other types of dendritic morphology were observed in the slices: Purkinje cells bearing either multipolar dendritic processes, or a single elaborated dendritic processes [28], [29]. Numerous branches and spines were detected on these 2 types of Purkinje cells and they corresponded to mature Purkinje cells. Interestingly, these two types of morphology were observed in all 3 conditions, i.e. control, BrdU-treated, and axotomy-treated slices (Fig. 3A3, B3, C3). Furthermore, it is known that Purkinje cells initiate their expression of Parvalbumin in parallel to their dendritic differentiation [30], [31]. In the three types of slices, control, BrdU-treated, and axotomy-treated slices, about half of the Purkinje cells were Parvalbumin immunoreactive after 14 DIV (Fig. 3A3, B3, C3). From these data, we can conclude that the different types of treatments do not affect two important parameters of Purkinje cell differentiation, i.e. dendritic differentiation and parvalbumin expression.

Thus, the use of conditioned medium taken from BrdU treated slices (BrdU-CM) allowed us to study the effect of slice cultures containing high numbers of differentiated Purkinje cells and no MBP+ oligodendrocytes. Whereas taking the CM from the “axotomy” slices (Axt-CM) allowed us to study of the effect of slice cultures containing very few differentiated Purkinje cells.

**Figure 4:**
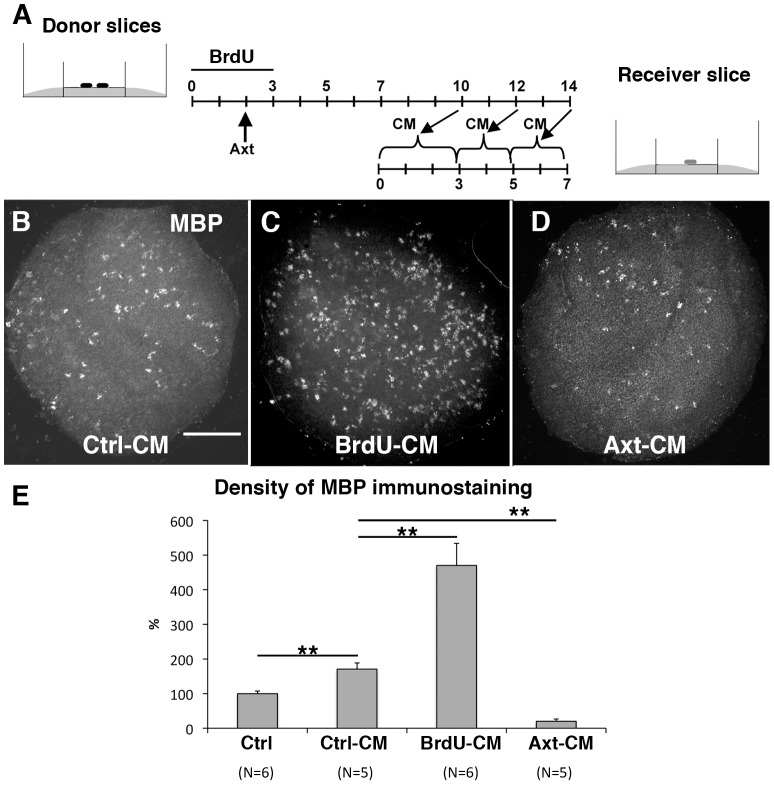
The effects of CM (conditioned media) from mature slices on OPC differentiation in immature slices are correlated with the presence of Purkinje cells. **A.** Schematic diagram of the co-culture experiment: P0 donor slices were grown for 14 days. The medium was changed at 3, 5, 7, 10, 12, and 14 DIV. The CM taken at 10 DIV was used for the culture of fresh recipient P0 slices from days 0 to 3; the CM taken at 12 DIV was used to culture recipient slices from days 3 to 5, and the CM taken at 14 DIV was used to culture recipient slices from days 5 to 7. **B.** The recipient P0–7 DIV slices grown in the presence of Ctrl-CM contained many MBP+ elements. **C.** The recipient P0 slice grown with BrdU-CM contains more MBP+ elements than that grown with Ctrl-CM (compare B and C). **D.** The recipient P0 slice grown with the Axt-CM contains few MBP+ elements (compare B and D). **E.** Quantitative evaluation of the density of MBP immunostaining in recipient P0-7DIV slices. Conditions: Ctrl corresponds to control P0–7DIV slices. Ctrl-CM corresponds to P0–7 DIV slices grown with the medium conditioned by untreated P0 7–14 DIV mature donor slices. BrdU-CM corresponds to P0–7 DIV slices grown with the medium conditioned by P0 7–14 DIV mature donor slices treated, for the first 3 DIV, with a high dose of BrdU. Axt-CM corresponds to P0–7 DIV slices grown with the medium conditioned by P0 7–14 DIV mature donor slices axotomized after 2 DIV. ** P<0.01 (Mann-Whitney tests). Scale bar is 390 µm for all images.

For P0 slices grown for seven days in the presence of conditioned medium from P0 7DIV–14DIV cultures (Ctrl-CM, Fig. 4A), the density of MBP immunostaining was significantly higher than in control slices, (1.72 times; Fig. 4E). Although this difference was smaller than for co-cultures, the obtained results show that mature slices release diffusible factors that increase oligodendrocyte differentiation in immature slices and that OPCs do not migrate from the mature to immature slices across the Millicell membrane.

We then tested the CMs from the two different culture conditions described above to manipulate the number of Purkinje cells. In all experimental conditions, we did not detect slices in Group II (i.e. presenting a significant number of internodes). Thus, the density of MBP might be used as an index of oligodendrocyte differentiation. P0 slices grown with BrdU-CM (Fig. 4C) had a density of MBP immunostaining that was 2.75 times higher than that in P0 slices grown with Ctrl-CM (Fig. 4B, E). In contrast, P0 slices grown with Axt-CM (Fig. 4D) had a very low density of MBP immunostaining which was 8.47 times smaller than P0 slices grown with Ctrl-CM (Fig. 4B, E). These findings suggest that BrdU-CM contained larger amounts of oligodendrocyte differentiation factors than Ctrl-CM, whereas Axt-CM contained less of these factors. This is consistent with a role for Purkinje cells as they are the only cell type whose numbers strongly changed in the two conditions, i.e. a large increase in BrdU treated slices and a large decrease in axotomy treated slices (see discussion). During their maturation, Purkinje cells therefore release or promote the release of factors stimulating oligodendrocyte differentiation.

### Sonic Hedgehog gene expression in the cerebellar cortex decreases during the first 10 postnatal days whereas that of vitronectin increases

**Figure 5:**
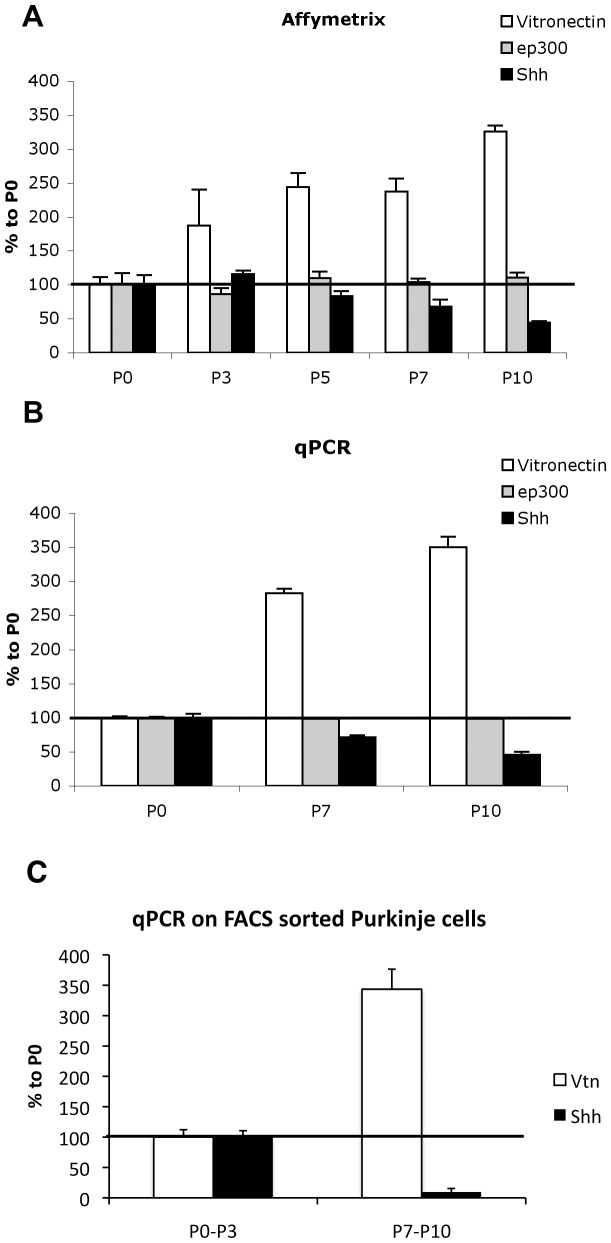
Sonic Hedgehog expression decreases and vitronectin expression increases during development. **A and B.** Temporal expression of vitronectin, ep300, and Shh mRNAs in cerebellar cortices enriched in Purkinje cells by Affymetrix (A) and qPCR (B) during the first 10 postnatal days. Values for P3, P5, P7, and P10 are expressed as a percentage of P0 values. Note the opposing developmental changes in the expression of Shh and VN, whereas the expression of ep300 does not vary. **C.** Expressions of Shh and vitronectin by RT-qPCR from L7-GFP purified PCs on a FACsAria apparatus during the first 10 postnatal days. Values for P0 and P3, and P7 and P10 are pooled respectively; P7–P10 values are expressed as a percentage of P0–P3 values.

To understand how Purkinje cells could be involved in the timing of oligodendrocyte differentiation, we looked for genes which are: regulated during postnatal development, expressed by Purkinje cells and are coding for extracellular proteins. We used Affymetrix arrays for transcriptome analysis on cerebellar cortical regions, at time points P0, P3, P5, P7, and P10. Statistical and gene ontology analyses identified 4 extracellular proteins encoded by genes displaying an expression that decreased, and 6 displaying an expression that increased by more than twofold during the first 10 postnatal days (see Table 1). Two of these, Sonic Hedgehog (Shh) and vitronectin, were of interest to us for various reasons; in particular the proteins of both are present in Purkinje cells [32]–[36]. Furthermore, Sonic Hedgehog (Shh) acts as a mitogen for OPCs [37], [38] and vitronectin overrides the proliferation response of granule cell progenitors to Shh, leading to the initiation of differentiation [39]. We confirmed the affymetrix expression patterns of Shh and vitronectin by qPCR (compare Fig. 5A and 5B).

**Table 1:** Selected gene candidates.

Affymetrix ID	Gene Symbol	P0/P0	P3/P0	P5/P0	P7/P0	P10/P0	Expression Pattern
1450716_at	Adamts1	1.000	0.825	0.679	0.599	0.364	decreased
1426670_at	Agrn	1.000	0.783	0.772	0.616	0.409	decreased
1451119_a_at	Fbln1	1.000	1.172	0.806	0.720	0.412	decreased
1436869_at	Shh	1.000	1.158	0.843	0.673	0.421	decreased
1424186_at	Ccdc80	1.000	1.644	3.097	4.046	4.829	increased
1449581_at	Emid1	1.000	1.816	1.848	1.993	2.525	increased
1424010_at	Mfap4	1.000	1.344	1.982	2.699	2.063	increased
1417678_at	Mmp24	1.000	0.980	1.367	1.374	2.041	increased
1451342_at	Spon1	1.000	2.148	2.245	2.652	3.499	increased
1420484_a_at	Vtn	1.000	1.640	2.284	2.717	3.334	increased

Expressions of the 10 transcription factors belonging to GO Cellular Component term ‘extracellular region/space’ (GO:0005576) and presenting a significant increase or decrease of expression measured by Affymetrix from cerebellar cortices during the first 10 postnatal days. Values for P0, P3, P5, P7, and P10 are expressed as a ratio of P0 values.

Then to check the expression patterns of Shh and vitronectin in developing Purkinje cells, we performed real-time quantitative PCR using mRNAs extracted from fluorescent flow cytometry sorted (FACS) Purkinje cells of L7-GFP transgenic mice at different ages (P0, P3, P7 and P10). In these experiments, the results obtained from P0 and P3 Purkinje cells were pooled together and compared to those of pooled P7 and P10. From this comparison, it clearly appeared that over the ten first postnatal days Shh presented a decreased expression, whereas vitronectin displayed an increased expression in Purkinje cells (Fig. 5C).

As Shh and vitronectin were good candidate modulators of the timing of cerebellar oligodendrocyte differentiation, we analyzed their roles in the control of the OPC proliferation/differentiation balance in organotypic cultures.

### Shh stimulates OPC proliferation and inhibits oligodendrocyte differentiation in organotypic culture

**Figure 6:**
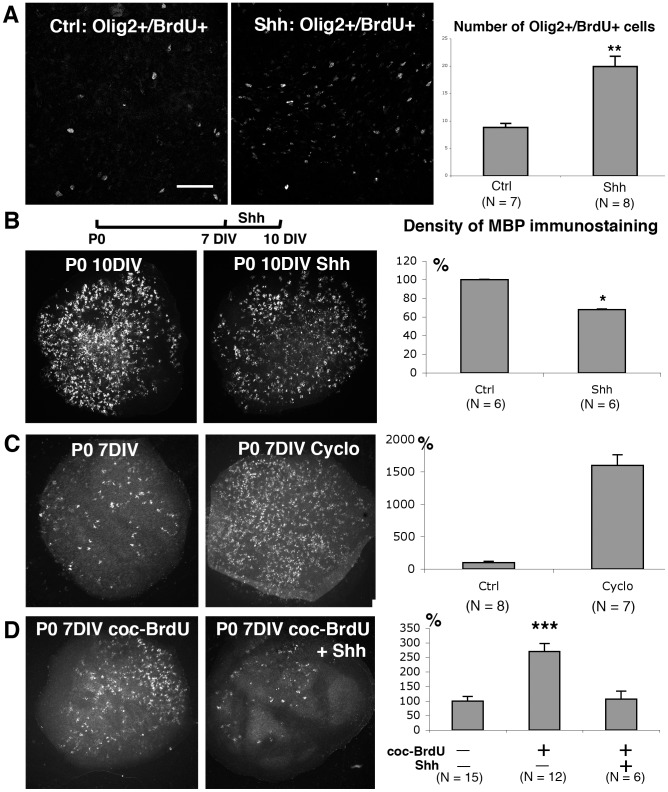
Role of Sonic Hedgehog in OPC proliferation and differentiation. **A: Proliferation of OPCs in response to Shh.** The proliferative capacity of OLIG2+ cells was assessed in the presence or absence of Shh. Images containing pixels labeled for both OLIG2 and BrdU were generated using the “AND” function of Metamorph software. Thus, these labeled cells were counted in presence or absence of Shh. OLIG2+ cell-proliferation rates were higher in the presence of Shh, as illustrated by the histogram. ** P<0.01 (Mann-Whitney test). **B: Differentiation of OPCs in response to Shh.** Diagram of Shh treatment schedule: P0–10 DIV mature slices cultured in the presence of Shh for the last 3 days of culture, i.e. between the 7th and 10th DIV. Photomicrographs of P0 cerebellar slices maintained for 10 DIV (mature) and labeled with antibodies against MBP. The MBP+ elements are less numerous in the slices treated with Shh for the last 3 days of culture (right) than in the control slice (left). Quantification of the density of MBP immunostaining (representing both oligodendrocytes and internodes) in P0–10 DIV mature control slices (Ctrl) and P0–10 DIV slices treated with Shh (Shh). * P<0.05 (Mann-Whitney test). **C: OPC differentiation in response to cyclopamine.** Photomicrographs of P0 cerebellar slices maintained for 7 DIV (immature) and labeled with antibodies against MBP. In the control slice (P0–7 DIV), the density of MBP immunostaining is low. In contrast, the density of MBP immunostaining is much higher in P0–7 DIV slices cultured in the presence of cyclopamine (P0–7 DIV Cyclo), a Shh inhibitor. Quantification of MBP immunostaining density in control (Ctrl) and cyclopamine-treated P0–7 DIV (Cyclo) immature slices. The density of MBP immunostaining was determined. ** P<0.01 (Mann-Whitney test). **D: OPC differentiation in co-culture experiments in the presence or absence of Shh.** Photomicrographs of P0 cerebellar slices labeled with antibodies against MBP at 7 DIV. These slices were co-cultured in the presence of P0 7–14 DIV mature slices treated with a high dose of BrdU during the first 3 days of culture (coc-BrdU) in the presence or absence of Shh. In the presence of Shh, the density of MBP immunostaining present in this immature slice was similar to that of control P0–7 DIV slices (compare C left and D right). In contrast, in the absence of Shh, there were more MBP+ elements in P0–7 DIV slices co-cultured with BrdU treated mature slices (coc-BrdU, left). Quantification of the density of MBP immunostaining in P0–7 DIV immature slices co-cultured in the presence or absence of BrdU treated mature slices and/or Shh. *** P<0.001 (Mann-Whitney tests). Scale bar in A is 70 µm for A and 425 µm for B, C, and D.

We investigated the effects of Shh treatment on OPC proliferation in P0–7DIV cultures by counting the OPCs (OLIG2+) incorporating BrdU following a short pulse. Treatment with Shh increased the number of OLIG2+/BrdU+ cells around Purkinje cell axons close to deep nuclear neuron regions (see Fig. 6A). Indeed, 8.8 cells per 150,000 µm2 were observed for control slices, whereas we observed 19.9 cells per 150,000 µm2 in the presence of Shh. Thus, Shh stimulates OPC proliferation in cerebellar P0–7 DIV slices.

We then investigated whether Shh inhibited OPC differentiation. We added Shh to the culture medium between the 7th and 10th days of culture (Fig. 6B) during a period of strong oligodendrocyte differentiation (compare Fig. 1A with 1B). In the presence of Shh, the density of MBP staining was smaller than in control P0–10DIV slices (Fig. 6B). Concerning the internodes, the presence of Shh on the P0–10 DIV slices did not significantly influence the percentage of slices in group II (presenting more than 26 internodes). The presence of Shh therefore slowed the rate of oligodendrocyte differentiation.

We then investigated whether Shh inhibition promoted oligodendrocyte differentiation. We added cyclopamine —an alkaloid known to block the Shh signaling pathway [40] — to the culture medium of P0 cerebellar slices during the first seven days of culture. This treatment greatly increased the density of MBP staining (Fig. 6C). However, cyclopamine did not affect the rate of myelination since all of the slices were in Group I (presenting less than 25 MBP+ internodes). These results indicated that inhibition of Shh induced an increase in oligodendrocyte differentiation but did not affect myelination.

Finally, we investigated whether Shh blocked oligodendrocyte differentiation in immature slices induced by the factors released by mature slices. We used BrdU-treated mature slices (depleted of OPCs; Fig. 3B) to prevent biases due to the possible effects of Shh on OPCs from the donor slices. We added Shh during the co-culture period. The density of MBP staining increased in P0–7DIV immature slices co-cultured with BrdU treated mature slices (Fig. 6D). In contrast, the density of MBP immunostaining in P0–7DIV immature slices co-cultured with 3DBT mature slices in the presence of Shh was similar to that in control immature P0–7DIV slices (Fig. 6D). Thus, in the presence of Shh, mature slices cannot stimulate oligodendrocyte differentiation in immature slices.

These results suggest that Shh prevents OPCs from exiting the cell cycle and inhibits the effects of oligodendrocyte differentiation factors —released by mature slices— on immature slices.

### Vitronectin promotes oligodendrocyte differentiation in organotypic culture

**Figure 7:**
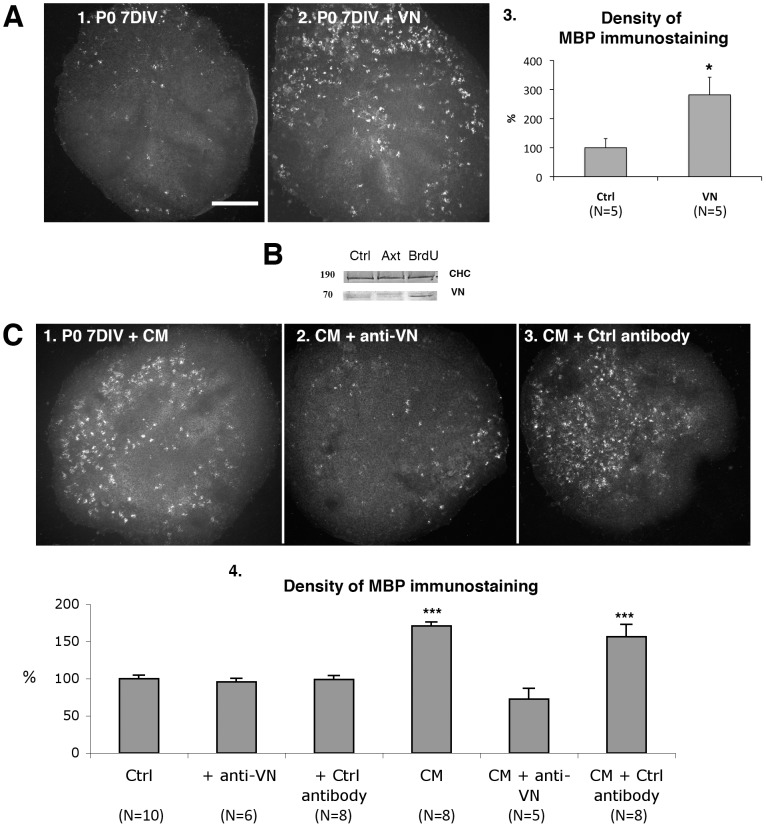
Role of vitronectin in oligodendrocyte differentiation. **A: Vitronectin increased oligodendrocyte differentiation.** A1–2. Photomicrographs of P0 cerebellar slices labeled with antibodies against MBP at 7DIV. The density of MBP+ elements in the control slice P0–7 DIV (A1) was lower than that in the slice grown in the presence of vitronectin (P0–7 DIV+VN, A2). A3. Quantification of MBP immunostaining in P0–7DIV immature slices. * P<0.05 (Mann-Whitney test). Ctrl (control slices), and VN (P0–7 DIV grown in the presence of vitronectin). **B: Immunoblots revealing the presence of vitronectin.** Immunoblot of vitronection (VN) in total lysates from P0–10 DIV cerebellar slices: control (Ctrl) or 2 DIV axotomy (Axt) or BrdU treated (BrdU) slices. Note the increase of vitronectin in BrdU treated slices in comparison to control and axotomized slices. The clathrin heavy chain (CHC) has been used as a loading control. **C:** Photomicrographs of P0 cerebellar slices labeled with antibodies against MBP at 7 DIV. C1. P0–7 DIV slice grown in the presence of CM (conditioned medium, as explained in Fig. 2). C2. P0–7 DIV slice grown in the presence of CM and an antibody against VN. C3. P0–7 DIV slice grown in the presence of CM and a control antibody. C4. Quantification of the density of MBP immunostaining in P0–7 DIV immature slices grown under various conditions: Control P0–7 DIV slices (Ctrl). In the presence of a blocking antibody against vitronectin (+ anti-VN). In the presence of a control antibody (+Ctrl antibody). With medium conditioned by P0 7–14 DIV control mature slices (CM). With medium conditioned by P0 7–14 DIV mature slices and a blocking antibody against vitronectin (CM+anti-VN). With medium conditioned by P0 7–14 DIV mature slices and a control antibody (CM+Ctrl antibody). *** P<0.001 versus the control (Mann-Whitney tests). Scale bar in A1 is 418 µm in A1–2 and 375 µm in C1–3.

We investigated the effects of vitronectin on immature slices. Addition of vitronectin (5 µM) during the first 7 DIV increased the density of MBP staining on P0–7DIV slices (Fig. 7A).

To affect oligodendrocyte differentiation, the extracellular matrix protein vitronectin must be released from cerebellar slices. Experiments with serum-free medium were also performed (see materials and methods) to test for the effects of soluble vitronectin in the medium. In co-culture experiments in serum-free medium, mature slices also significantly promoted oligodendrocyte differentiation in immature slices (*** P<0.001 Mann-Whitney test; data not shown).

We also compared the amounts of vitronectin in control, axotomized and BrdU treated P0–10DIV slices. As expected, we observed more vitronectin in BrdU treated slices than in control and axotomized slices (Fig. 7B). We detected only a slight difference in the amount of vitronectin between control and axotomized slices (Fig. 7B). These results demonstrate that increasing the number of Purkinje cells increased the amount of vitronectin.

Finally, we used blocking antibodies to determine whether vitronectin present in the CM induced the increase in OPC differentiation. In the presence of these antibodies, the CM did not promote OPC differentiation in immature slices (Fig. 7C2, 7C4), whereas the CM alone (Fig. 7C1, 7C4), or in the presence of a control antibody, promoted OPC differentiation (Fig. 7C3, 7C4).

Our findings suggest that Sonic Hedgehog and vitronectin play important antagonistic roles in controlling the timing of OPC differentiation during cerebellar development.

## Discussion

Different cell types differentiate in parallel during development. The synchronization of cell development is particularly important for cell types that have strong interactions, such as oligodendrocytes and neurons. OPCs start to differentiate if a mitogenic stimulus is removed or a differentiation stimulus is added. This cell differentiation is inhibited in the presence of a mitogenic stimulus and the absence of a differentiation stimulus [17]. After exiting the cell cycle, OPCs become immature oligodendrocytes which undergo different phases of differentiation [1], [23]. The last two phases of oligodendrocyte differentiation are the production of myelin proteins and myelination. In the present study, we found that Purkinje cells control the timing of oligodendrocyte differentiation in cerebellar organotypic culture by releasing diffusible factors. Indeed Shh and vitronectin, two proteins present in the extracellular space and expressed by Purkinje cells, had opposite patterns of expression during development and showed opposing effects on oligodendrocyte differentiation.

### Role of mature Purkinje cells in oligodendrocyte differentiation in organotypic cultures

Mature slices promoted oligodendrocyte differentiation in immature slices. We modified the number of Purkinje cells present in the slices to investigate whether this cell type is involved in the stimulation of oligodendrocyte differentiation during the second week of culture. We have previously reported, that high doses of BrdU treatment during the first three days of culture generates cerebellar slices with large numbers of Purkinje cells and reactive astrocytes, but with markedly decreased numbers of oligodendrocytes and microglial cells [27]. Furthermore, we have not detected any changes in other neuronal population, such as granule cells and molecular interneurons [27]. The increase in Purkinje-cell number in BrdU-treated slices may be due to the depletion of microglial cells, which enhances Purkinje cell survival in organotypic culture [41].

In the present study, CM from mature slices treated with BrdU had a stronger effect on oligodendrocyte differentiation than CM from control mature slices. The almost complete absence of oligodendrocytes and microglial cells from mature slices treated with BrdU rules out the self-stimulation of oligodendrocyte differentiation [18], [42] and the release of factors from microglial cells. The present study therefore implies a role for Purkinje cells or astrocytes. The use of CM from axotomized mature slices favors a role for Purkinje cells and not for reactive astrocytes. Indeed, no oligodendrocyte differentiation was observed in slices treated with CM from axotomy treated slices, despite the persistence of reactive astrocytes in the manipulated slices (data not shown). However, we cannot exclude that the reactivity of astrocytes is different between BrdU and axotomy experiments and therefore that they release different molecules under these two conditions, as recently reported in a different context [43].

Altogether our findings indicate that among the cells in mature slices, Purkinje cells and astrocytes might be responsible for directly or indirectly releasing factors promoting oligodendrocyte differentiation in immature cultures. We verified through the analysis of two parameters (dendritic morphology and expression of parvalbumin) that Purkinje cell differentiation occurred after the two types of treatment (BrdU and axotomy).

### During maturation, Purkinje cells down regulate the expression of Shh and up regulate the expression of vitronectin

We then hypothesized that during their maturation Purkinje cells might express differentially genes to synchronize OPC differentiation with their own differentiation. Transcriptome analysis performed on cerebellar cortex during the first postnatal week showed a decreasing expression profile of 4 genes and an increasing expression profile of 6 genes. Interestingly, among them, Shh and vitronectin were of special interest. Shh is well known to be produced by Purkinje cells [32]–[35], [39] and is a mitogen for OPCs [37], [38]. Interestingly, Gupta and colleagues showed that vitronectin is expressed in Purkinje cells at P0 and this expression is upregulated at P8 [36]. Last, vitronectin overrides the proliferation response of granule cell progenitors to Shh, leading to the initiation of their differentiation [39].

The transcriptome analysis has been performed on cerebellar cortex. Nevertheless, the same profiles were observed for Shh and vitronectin in GFP expressing PCs sorted by FACS, demonstrating that during their maturation Purkinje cells down regulate the expression of Shh and up regulate their vitronectin expression. Thus, Purkinje cells might indeed control the differentiation of OPC by regulating the expression of Shh and vitronectin during their differentiation.

### Role of Shh and vitronectin in oligodendrocyte differentiation in organotypic cultures

Then, we checked whether Shh and vitronectin were able to participate to the control of OPC differentiation process in organotypic culture.

We showed that Shh stimulated proliferation of OPCs and inhibited oligodendrocyte differentiation in our cultures. Shh affects OPC in different ways. First, Shh plays an important role in OPC specification in the spinal cord [44]–[46] and brain [47]–[51]. Second, Shh stimulates OPC proliferation [37], [38], [52]. Our results confirm the mitogenic effect of Shh on OPCs. These results also suggest that changes in Shh expression may control the timing of OPC differentiation (see below). Thus, Purkinje cells, through Shh secretion, participate in the control of OPC proliferation in the cerebellum.

The addition of vitronectin to immature slices enhanced oligodendrocyte differentiation and vitronectin-blocking antibodies inhibited the effects of mature-slice induced differentiation on immature slices. Furthermore, when the slices cultures contained more Purkinje cells (following BrdU treatment), levels of vitronectin were elevated. Only a slight difference between control and axotomized slices was detected likely because we are closed to the threshold of detection. Thus, our results clearly demonstrate that vitronectin is required for mature slices to promote oligodendrocyte differentiation in immature slices. Previous studies reported no effect of vitronectin on OPC differentiation [53]. Moreover, it has been shown that the CG4 cell lines grown on vitronectin proliferate more that those grown on polylysine [54]. However, combinations of vitronectin and mitogenic factors (Shh, retinoic acid, and noggin) stimulate the differentiation of embryonic stem cells into oligodendrocytes, whereas vitronectin alone has no effect [55]. Interestingly, Vitronectin inhibits the effects of Shh in other systems, through biochemical interactions in the spinal cord [56] or the signaling pathway induced by its binding to integrin in cerebellar granule cells [39], [57]. Nevertheless, Shh and vitronectin may also control OPC differentiation through different mechanisms.

### The oligodendrocyte differentiation is regulated temporally by multiple factors in an integrated system: the importance of orchestration

Organotypic culture is an integrated system, in which cell interactions mimic those occuring in vivo, and is easier to manipulate than in vivo models. Furthermore only one type of neuron is myelinated in this system: the Purkinje cell. Using this system, we showed that the maturation of the Purkinje cell is involved in controlling the timing of oligodendrocyte differentiation. Indeed, our results suggest that Purkinje cells release different factors during their maturation, which have opposing effects on oligodendrocyte differentiation. This temporal regulation probably synchronizes the differentiation of oligodendrocytes and Purkinje cells. However, as discussed above, oligodendrocyte differentiation occurs even when the number of Purkinje cells is reduced (axotomy experiment). This suggests the presence of other differentiating factors in cultured slices or medium. Many of the factors known to affect oligodendrocyte development, such as TGFß, IGF-1, and progesterone [1], are present in the cerebellum [58]–[63]. Oligodendrocyte differentiation rates were lower in serum-free medium than in the presence of serum. Thyroid hormones (TH) are crucial for both oligodendrocyte and Purkinje cell differentiation [1], [64]. Among the factors involved in controlling OPC proliferation and differentiation, our study identifies two molecules with developmentally regulated expression patterns which are involved in the switch between OPC proliferation and oligodendrocyte differentiation. Vitronectin has been detected in active multiple sclerosis plaques [65] and a decrease in Shh levels has been observed in the white matter of patients with multiple sclerosis [66]. These actors are thus present, but it is unclear as to whether they are correctly synchronized. Determining how the timing of the various steps leading to myelination or remyelination is controlled is therefore still a major challenge.

The findings of the present study strongly imply that Purkinje cells initially inhibit OPC differentiation during their maturation by releasing Shh and then subsequently promote OPC differentiation by producing vitronectin. Purkinje cells thus appear to orchestrate OPC differentiation in cerebellar organotypic cultures.

## Materials and Methods

### Slice culture

All procedures were submitted and approved by the Regional Ethics Committee in Animal Experiment N°3 of Ile-de-France region (p3/2009/020). Cerebellar organotypic cultures were established from newborn (P0) Swiss mice (Mus musculus, Janvier, Le Genset St Isle, France), as previously described [67]. Mice were decapitated and their brains were dissected out in cold Gey's balanced salt solution (Invitrogen, Cergy Pontoise, France) supplemented with 5 mg/ml glucose. Cerebellar parasagittal slices (350 µm thick) were cut on a McIlwain tissue chopper and transferred onto 30 mm diameter Millipore culture inserts with 0.4 µm pores (Millicell, Millipore, Molsheim, France). Slices were maintained in culture in six-well plates containing 1 ml of nutrient medium per well, at 35°C, under a humidified atmosphere containing 5% CO2. The nutrient medium consisted of 50% basal medium with Earle's salts (BME, Invitrogen), 25% Hanks' balanced salt solution (Invitrogen), 25% horse serum (Invitrogen), L-glutamine (1 mM), and 5 mg/ml glucose [68].

For each result, At least 5 animals and 25 slices were studied in 3 independent experiments. In the figures, “N” is the number of animals. The experimental plan was designed in accordance with the European Union Guidelines for the care and use of experimental animals.

In experiments evaluating the effect of a decreased number of Purkinje cells, tissue slices were dissected along the midline between the dorsal and ventral halves, with two needles, under a dissection microscope, after 2 days in vitro (DIV). The two parts were gently separated to ensure complete axotomy and were then re-apposed.

### Exposure of cerebellar slices to Sonic Hedgehog, cyclopamine, vitronectin, anti-vitronectin antibody, or 5-bromo-2-deoxyuridine

We added Shh (3 µg/ml, R&D Systems, Lille, France), cyclopamine (5 µM, Toronto Research Chemicals, North York, Ontario, Canada), vitronectin (VN; 5 µM, Oxford Chemical Research, Euromedex, Souffelweyersheim, France), goat anti-VN antibody (0.2 µg/ml, Abcam, Cambridge, UK), or a control goat antibody (goat anti-Brn3b, 0.2 µg/ml, Santa Cruz Biotechnology, Santa Cruz, CA) to the medium. The medium and the added drug were replaced every two to three days. Untreated slices were used as controls.

Cerebellar slices were depleted of mature oligodendrocytes by killing OPCs through the addition of 5-bromo-2-deoxyuridine (BrdU; 150 µM; Sigma, Saint Louis, MO) in NaCl solution (9 g/l) to the nutrient medium for the first 3 DIV (P0 slices with a 3-day BrdU treatment: P0–3DBT [27].

To study the effect of Shh on OPC proliferation, both control and treated (5 µM Shh) slices were incubated with BrdU (20 µM) for 3 h before fixation.

### Co-culture and conditioned medium (CM) experiments

P0 slices maintained in culture until 7 DIV contained very few MBP (myelin basic protein) immunoreactive oligodendrocytes (Fig. 1A, A′), whereas culture of these slices for an additional 3 DIV (10 DIV in total) resulted in the accumulation of MBP immunoreactive oligodendrocytes and few MBP immunoreactive internodes (myelin segments, Fig. 1B, B′). Seven DIV was therefore used as the cutoff to distinguish between “immature slices” and “mature slices”. P0 slices were placed in culture and, when these slices became mature after 7 DIV, fresh P0 slices were added (immature).

A slice ratio of 2 matures: 1 immature (Fig. 2A) was used to assess whether mature slices promoted oligodendrocyte differentiation in immature slices. Co-cultures were incubated for 7 DIV before fixation.

In some experiments, immature slices were cultured on conditioned medium (CM). CM was obtained from cultures of P0 slices, between the 8th and the 14th days of culture (Fig. 4A). Different types of CM were generated by treating P0 slices to modify the number of Purkinje cells; control P0 slices provided Ctrl-CM, BrdU treated slices provided BrdU-CM, and axotomized slices provided Axt-CM.

### Antibodies and staining procedure

Mouse monoclonal antibodies against MBP (diluted 1/500, Chemicon, MAB381, Millipore) were used to visualize mature oligodendrocytes and myelin [22], [27], [69]. Goat antibodies against OLIG2 (1/500 R&D Systems Europe, Abingdon, UK) were used to visualize OPCs and oligodendrocytes [26]. Mouse monoclonal antibodies against APC (1/1000, Calbiochem, VWR, Fontenay sous bois, France) were used in combination with anti-OLIG2 to detect oligodendrocytes. Mouse monoclonal antibodies against BrdU (1/400 Progen, Heidelberg, Germany) or were used to identify proliferating cells. Rabbit polyclonal antibodies against calbindin (Calb1; diluted 1/5,000; Swant, Bellinzona, Switzerland) and goat antibodies against Parvalbumin (1/5000, Swant) were used to visualize differentiated Purkinje cells [22].

After 7, 10, or 14 DIV, the cultures and co-cultures were fixed by incubation in 4% paraformaldehyde in phosphate buffer (0.1 M, pH 7.4) for 1 h at room temperature. Slices incubated with anti-MBP antibody were washed in PBS (Invitrogen) and immersed in Clark's solution (95% ethanol/5% acetic acid) for 20 min at 4°C to extract some of the lipids —increasing the accessibility of MBP antigens— and were then washed several times with PBS. All slices were incubated for 1 h in PBS containing 0.2% gelatin, 0.1% sodium azide (PBSGA) and 0.1 M lysine. This was followed by an overnight incubation at room temperature with primary antibody diluted in PBSGA. The primary antibodies were detected with the following secondary antibodies: CY3-conjugated goat anti-mouse (1/200 dilution; Jackson ImmunoResearch Laboratories Inc, West Baltimore Pike, PA), FITC-conjugated sheep anti-rabbit (1/200 dilution, Chemicon), CY3-conjugated donkey anti goat (1/200 dilution, Jackson Immunoresearch), Alexa Fluor 488-conjugated donkey anti mouse (1/400 dilution, Invitrogen), AMCA-conjugated donkey anti rabbit (1/50 dilution, Jackson Immunoresearch), CY3-conjugated goat anti-rabbit (1/200 dilution; Jackson ImmunoResearch). Slices incubated for 2 h with the secondary antibodies in PBSGA were washed several times in PBS and mounted in Mowiol (Calbiochem). The sections were analyzed under a Leica DMR microscope equipped with a Coolscan camera (Princeton Instruments, Evry, France).

### MBP immunostaining quantifications

MBP immunoreactive oligodendrocytes and internodes (myelin segments) were quantified by acquiring an image of each slice using the software, Metaview. The images were then analyzed using the software, Metamorph (Universal Imaging Corporation). Contours of the slices were drawn —the threshold was adjusted based on the MBP immunostaining— and the density of MBP immunostaining was determined for each slice. Means and standard errors of the mean (SEM) were calculated for each type of slice.

To distinguish between oligodendrocyte differentiation and myelination, we performed a semi-quantification of MBP+ internodes which did not consider the number of differentiated oligodendrocytes. The ×5 pictures were enlarged on the computer screen (zoom 4) to evaluate the number of internodes, and the first 26 internodes were pointed and counted using ImageJ software. Two groups were specified: Group I included slices that either did not contain MBP+ internodes (myelin segments) or contained less than 25 internodes; group II included slices containing more than 26 internodes.

### Quantification of OLIG2 positive and APC positive cells

On slides with Calb1 labeling of Purkinje cells, pictures of areas centered on white matter close to the deep nuclear neurons were acquired for OLIG2 and APC using the Metaview software. Then, number of cells positive for both OLIG2 and APC per 81,000 µm2 were determined manually using ImajeJ software. Means and standard errors of the mean (SEM) were calculated for each type of slice.

### Quantification of OLIG2-positive and BrdU-positive cells

A proliferation index for OPCs was calculated by determining the number of cells positive for both OLIG2 and BrdU. On slides with Calb1 labeling of Purkinje cells, single areas of white matter close to the deep nuclear neurons were chosen, 1 µm thick confocal sections were acquired for OLIG2 and BrdU labeling (Leica confocal microscope; Plateforme d'Imagerie, IFR83). Using the software Metamorph, images containing pixels labeled for both OLIG2 and BrdU were generated and the number of double-stained cells per 150,000 µm2 was determined.

### Statistic analysis

Mann-Whitney tests were used to compare two groups and Kruskal-Wallis tests were used for multiple comparisons. P values≤0.05 were considered to significant. In the figures, the values are represented as the percentage of the control.

### Statistical analysis of Affymetrix data, microarray annotation, and functional analysis

Affymetrix microarrays were used to assess patterns of gene expression in murine cerebellar cortical areas. Total RNA was extracted from dissected areas centered on the layer of Purkinje cells from vermal lobules 5 and 6 of Swiss mice of various ages: P0, P3, P5, P7, and P10 (4 replicates = 4 independent measurements for each stage; different litters were used for each measurement). RNA was extracted using RNAeasy Mini Kit (Qiagen, Courtaboeuf, France). Following reverse transcription, cDNAs were hybridized with Affymetrix microarrays (MOE430 GeneChip, Affymetrix Platform, Institut Curie, Paris, France). All the experimental procedures and results have been loaded on ArrayExpress database (E-MEXP-3444, Experiment name: PC transcriptome, http://www.ebi.ac.uk/arrayexpress/experiments/E-MEXP-3444).

Matlab was used for mathematical manipulations and statistical analysis. The aim was to identify transcripts displaying a significant increase or decrease over time from P0 to P10.

The four replicates were used to estimate the intrinsic variability of expression, or pure error, for each transcript. We used the pure error in F-tests of lack of fit [70] for constant, affine, and quadratic models. The Benjamini-Hochberg procedure [71] was used for correction, with estimation of the number of true null hypotheses [72] to guarantee an FDR of 1%.

A constant model was retained (i.e. no lack of fit) for 26,912 of the 31,818 transcripts, an affine model was retained (lack of fit of the constant model, but not of the affine model) for 4,474 transcripts, and a quadratic model was retained (lack of fit of the constant and affine models, but not of the quadratic model) for the remaining 432 transcripts. All “constant” transcripts were discarded. We retained all of the “affine” transcripts as potential candidates. We also searched for “quadratic” transcripts displaying continual increases or decreases. One quadratic transcript was identified with a continually decreasing pattern over time. Two lists were therefore generated: 2,262 affine transcripts showing continuous increases in expression and 2,213 transcripts with continuous decreases in expression (2,212 quadratic, 1 affine). Among the latter, we selected transcripts with at least a two-fold difference in expression (higher or lower) at P10 compared with P0. This gave a list of 1190 transcripts, from which we further discarded those with a very low level of expression (below 45), to give a final list of 627 candidate transcripts.

Gene annotations were expanded and upgraded with NCBI Entrez Gene ID, Unigene, and PubMed for the remaining 627 candidate probesets. Transcribed sequences and expression sequence tags (ESTs) with no identified function were eliminated from the reported lists. Gene annotations for the MOE430 GeneChip obtained from Webgestalt (web-based gene set analysis toolkit [73]) and from the Affymetrix website were compared. Gene ontology (GO) terms were applied and 10 genes were identified based on the GO cellular component term “extracellular region/space” (GO:0005576).

### Quantitative RT-PCR

RT-PCR quantified in real time was carried out on two independent sets of mRNA for each age (P0, P7, P10), to assess Shh and VN mRNA levels using a LightCycler 480 real-time PCR system (Roche). First-strand cDNAs were synthesized from 500 ng total RNA (Thermo Scientific, Surrey, UK), in accordance with the manufacturer's instructions. The reaction mixture contained 1× SYBR Green I Master (Roche Diagnostics, Mannheim, Germany), 1.5 ng of the first-strand cDNA and 240 nM of each forward and reverse primer, in a total volume of 13 µl. Thermal cycle parameters were: 95°C for 5 min, followed by 40 cycles of 95°C for 10 s, 56°C for 20 s, and 72°C for 20 s. All primers were synthesized by Eurofins MWG Operon (Ebersberg, Germany). The following primer sequences were used: VN forward: 5′-GCCAGCAAGAAGTGTCAGTG-3′, VN reverse: 5′-GCTTTAGAGTGCCGTCCGTC-3′, Shh forward: 5′-TCACCCCCAATTACAACCCC-3′, Shh reverse: 5′-ACTCCTCTGAATGATGGCCG-3′, Ep300 forward: 5′-GAGGGTAAGAAATGACTCCAGTG-3′, Ep300 reverse: 5′-CAGTTGAGGGTTTTTGCTTTC-3′. Data were analyzed by the LightCycler 480 software, release 1.4.9 (Roche), and target-transcript expression was quantified using the second-derivative maximum method. Control reactions without reverse transcriptase were performed to check for genomic DNA contamination. All samples were normalized with respect to the reference gene, Ep300, which displays minimal variation during the first 10 days of cerebellar development. Two independent experiments were used for quantification. Data are presented as means ± SEM.

### Purkinje cell sorting

Purkinje cells were isolated from BacL7-GFP mice as described previously [74]. Briefly, cubes (0.5-mm3) of cerebella at indicated ages were digested for 10 min at 37°C with 0.025% trypsin (type I; Sigma-Aldrich, Saint Louis, MO) in dissociation solution consisting of Ca2+-free Hanks' balanced salt solution (HBSS) containing 3 mg/ml BSA, 15 mM Hepes, 1.5 mM MgSO4, and 3 mg/ml glucose (pH 7.4). The enzymatic reaction was stopped by adding of one volume of dissociation solution containing 0.25 mg/ml soybean trypsin inhibitor, 40 mg/ml DNase I, 50 µM APV, 20 µM DNQX and 0.1 µM TTX (all from Sigma-Aldrich). Tissues were triturated mildly by sequential passage through wide-bore and fine-tipped pipettes. Cells were filtered through a 40 µm nylon mesh, and were resuspended in Ca2+- and Mg2+-free dissociation solution at a final concentration of 5.106 cells per ml. To label the dead cells, PI (Propidium iodide; Sigma-Aldrich) was added at a final concentration of 2 µg/ml. Cell sorting was performed on a FACsAria machine. The sorting decision was based on the measurements of FSC, PI fluorescence and GFP fluorescence.

### RNA preparation and real time quantitative RT-PCR for purified Purkinje cells

Total RNA was extracted from approximately 3.104 purified Purkinje cells at indicated stages with Trizol reagent (Invitrogen). The cDNA was prepared by reverse transcription of 100 ng RNA using SuperScript III First-Strand Synthesis System (Invitrogen) with an oligo-dT primer according to the manufacturer's instructions. The resulting cDNA was used as a template for real time PCR using a Light Cycler 480 thermocycler (384 plates, Roche Diagnostics) with a home-made SYBR Green QPCR master mix [75]. Thermal cycling parameters were 2 min at 95°C, followed by 45 cycles of 95°C for 10 s, 64°C for 15 s and 72°C for 25 s. To normalize expression data, multiple internal control genes (TATA box binding protein, Actin beta and Tubulin beta 2A) were selected as described by Vandesompele et al. [76]. Sequences of the primers used can be obtained upon request.

### Western blots

Cerbellar slices were lysed in RIPA buffer (50 mM Hepes, 150 MM NaCl, 5 mM EDTA, 1% NP-40, 0.5% SDS; pH 7.7). Lysates were clarified by centrifugation. The protein pellet was collected by centrifugation, washed with cold acetone and recentrifuged. The pellet was then allowed to air dry and resuspended in 10 mM Tris pH 8. The DCA protein assay (Biorad, Hercules, California) was used to determine protein levels. Samples (2 µg of CM proteins) were denatured by heating for 3 min at 95°C in Laemmli buffer, separated by 10% SDS-PAGE, and transferred to a polyvinylidene difluoride membrane (PVDF, Amersham Biosciences, GE Healthcare, UK). TBS supplemented with 0.1% Tween-20 and 5% dry milk powder was used for blocking and antibody incubations. Primary (rabbit polyclonal anti-VN, 1/1000) and secondary Alkaline phosphatase conjugated goat anti-rabbit polyclonal antibody (1/7500, Promega, Madison, WI). Membranes were incubated overnight at 4°C with the primary antibody and then for 1 h at room temperature with the secondary antibody. They were then washed three times in TBS supplemented with 0.1% Tween-20 and once in TBS. The alkaline phosphatase-conjugated antibody was detected by NBT/BCIP (Promega). A prestained protein ladder (Fermentas, Euromedex, Souffelweyersheim, France) was used as the size marker.
